# “Abbreviation Arrangement” Syndromes, i.e., Names and Natures Do Often Disagree

**DOI:** 10.31662/jmaj.2021-0209

**Published:** 2021-12-24

**Authors:** Michito Sadohara

**Affiliations:** 1 Department of Community, Family, and General Medicine, Kumamoto University Hospital, Kumamoto, Japan

**Keywords:** Clinical diagnostic reasoning, diagnostic criteria, SAPHO syndrome, abbreviation

A syndrome is referred to as an identified combination of constellational symptoms or signs displaying concurrently. The symptoms or signs may represent particular diseases or associated conditions that correlate with each other. The nomenclatures were made by various ways, e.g., eponymously (e.g., Guillain-Barre syndrome), by conditions (e.g., locomotive syndrome), by symptoms (e.g., chronic fatigue syndrome), by radiological features (e.g., RESLES, reversible splenial lesion syndrome), or pathological features (e.g., vasculitis syndrome), among others. Clinicians sometimes encounter the name of syndromes spelled with abbreviations arranged by initials, acronyms, acronyms, or mnemonics. The examples of these “abbreviation arrangement” syndromes are listed in the Table 1. Some syndromes are relatively rare. Some often involve multi-organ systems or present as a systemic disease. Most of them have descriptive definitions, diagnostic criteria, or classifications reflecting the abbreviations because the etiologies and a part of their pathogenesis are not well-known and monocausal. Nonetheless, once clinicians had known the original meaning of abbreviations and their clinical entities, the letters embedded in the syndromes can easily evoke the memory through pattern recognition as a fast, automatic, unconscious, heuristic, and intuitive process of System 1 decision-making in dual process theory ^(1)^, leading to an “Aha!” moment. Most abbreviations represent typical or unique symptoms, signs, or features, and some correspond to semantics by themselves, e.g., “remitting seronegative symmetrical synovitis with pitting edema (RS3PE) syndrome” comprises many semantics. Even when each feature of a syndrome is so common and not specific by itself alone, combining them together or regarding them as a round set will make them very specific, such as the Bayesian approach, to rule in to correct diagnosis.

**Table 1.** Examples of “Abbreviation Arrangement” Syndromes.

Abbreviations	Original word from each initial capital letter
HELLP syndrome	Hemolysis, Elevated Liver enzymes, and Low Platelet count
RS3PE syndrome	Remitting Seronegative Symmetrical Synovitis with Pitting edema
PFAPA syndrome	Periodic Fever, Aphthous Stomatitis, Pharyngitis, Adenitis
POEMS syndrome	Polyneuropathy, Organomegaly, Endocrinopathy, Monoclonal gammopathy, and Skin changes
SAPHO syndrome	Synovitis, Acne, Pustulosis, Hyperostosis, Osteitis
TAFRO syndrome	Thrombocytopenia, Anasarca, Fever, Reticulin fibrosis

However, some difficulty exists in diagnosing these syndromes. Since specific biomarkers or effective imaging modalities are sometimes not known or available, it results in empirical or diagnostic therapy. These concepts have been changed from the original by advocators along with advancing technology and enhanced accumulation of findings. For example, RS3PE syndrome was known as paraneoplastic syndrome and as prodrome of elderly onset rheumatoid arthritis. Pustulotic arthro-osteitis was incorporated with synovitis, acne, pustulosis, hyperostosis, and osteitis (SAPHO) syndrome. Crow-Fukase disease is now regarded to have the same concept as polyneuropathy, organomegaly, endocrinopathy, monoclonal gammopathy, and skin changes (POEMS) syndrome. Thrombocytopenia, anasarca, fever, and reticulin fibrosis (TAFRO) syndromes are considered a distinct variety of human herpes virus 8 (HHV-8)-negative multiple Castleman’s disease. Sometimes, some abbreviations embedded in the syndromes are the shells of their former selves. Unilateral PS3PE is well recognized, and titer of anti-matrix metalloproteinase-3 (anti-MMP-3) antibody can often increase, otherwise rheumatoid factor and anti-cyclic citrullinated peptide (anti-CCP) antibody are negative ^(2)^. Although the diagnostic criteria define a lot of exclusives, the inclusion criteria may not be strict, and whichever are applicable, resulting in deterioration for diagnostic specificity.

It is not surprising if “SAPHO syndrome without cutaneous lesions” was made as the case report ^(3)^, since clinicians recollected the osteoarthral presentations of SAPHO syndrome at a very early stage before the cutaneous lesions emerged. In fact, skin involvements antedate to musculoskeletal symptoms in about 40%-70%, less than 30% occur concurrently, followed by about 30%-60%, and at least 15% are free from skin lesions ^(4)^. A bull’s head sign is so specific that the diagnosis of SAPHO syndrome cannot be denied, even if not all components were concurrently present or if diagnosis was not met in some criteria.

There is an increased prevalence of patients recognized and diagnosed, and many reports through relevant cases, case series, cohorts, and registered patients to follow up are increasing. These findings will contribute to the redefinition and revisions of diagnostic criteria, discovery of etiologies, true prevalence, consequences, and therapeutic measures or options for these syndromes. Advances in radiological technologies made the diagnosis more effective. Various modalities, including ultrasonography with color Doppler, positron emission tomography, and other radioactive scans, can be used for musculoskeletal pathologies. Vascular endothelial growth factor is now a major contributor for polysynovitis and edema of RS3PE syndrome and POEMS syndrome. Immunomodulators and biologics are growing therapeutic options along with disease-modifying agents, immunosuppressive drugs, and steroids.

For clinical diagnostic reasoning, from patients’ presentations of illness through comparing and contrasting the features of the defining and discriminating features of illness, disease, or syndromes, the process of retrieval of accumulated illness scripts as representation from the knowledge network comprising the predisposing conditions, pathophysiological insults, and clinical consequences is important ^(5)^. A schematic diagram of five symptoms and signs of SAPHO syndrome and related diseases is shown in the Figure 1. Regardless of these scattered symptoms, signs, and diseases with complexity, the set of abbreviations makes it easier for clinicians to retrieve the illness scripts for SAPHO syndrome. Reviewing and verifying each feature is difficult as the diagnosis can lead to different names depending on the context and framework from different viewpoints even though the names of diagnoses represent the same meanings.

**Figure 1.**
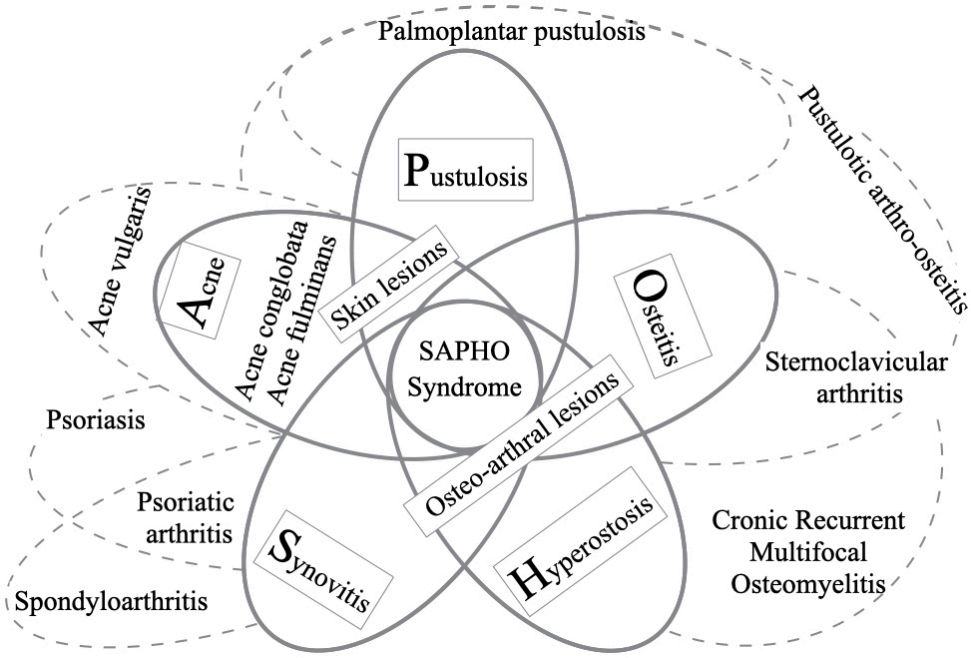
Five symptoms and signs of SAPHO syndrome and related diseases. Note: Overlapping areas indicate defining features, non-overlapping areas indicate discriminating features of the syndrome.

More evidence-based approach will be needed for more accurate diagnosis, to verify the abbreviations, estimating as if they are variables weighted on the entities of the syndrome or clinical prediction rules. Therefore, further evidence-based consensus and revisions for diagnostic criteria must be developed for these syndromes. Some diseases will be incorporated into these syndromes, while others will remain within the original framework owing to their convenience or be isolated to a syndrome “without (w/o) some abbreviation.” Nevertheless, further investigations into etiopathogenesis are warranted to reveal the entities of these syndromes and to establish definite concepts and criteria for diagnosis.

## Article Information

### Conflicts of Interest

None

### Author Contributions

Author M.S. conceived the idea and the concept of this article and led the manuscript.

### Approval by Institutional Review Board (IRB)

Not applicable because this article is an editorial article.
